# Challenges and Lessons Learned in the Development of a Participatory Learning and Action Intervention to Tackle Antibiotic Resistance: Experiences From Northern Vietnam

**DOI:** 10.3389/fpubh.2022.822873

**Published:** 2022-07-26

**Authors:** Huong Thien Ngoc Cai, Hang Thi Tran, Yen Hong Thi Nguyen, Giao Quynh Thi Vu, Thao Phuong Tran, Phuong Bich Bui, Huong Thi Thu Nguyen, Thai Quang Pham, Anh Tuan Lai, Jennifer Ilo Van Nuil, Sonia Lewycka

**Affiliations:** ^1^Oxford University Clinical Research Unit, Hanoi, Vietnam; ^2^Communicable Disease Control and Prevention, National Institute for Hygiene and Epidemiology, Hanoi, Vietnam; ^3^Centre for Disease Control, Nam Dinh, Vietnam; ^4^Centre for Tropical Medicine and Global Health, Nuffield Department of Medicine, University of Oxford, Oxford, United Kingdom

**Keywords:** AMR, PLA, community, participation, partnership, trust, engagement, Vietnam

## Abstract

Antibiotic use in the community for humans and animals is high in Vietnam, driven by easy access to over-the counter medicines and poor understanding of the role of antibiotics. This has contributed to antibiotic resistance levels that are amongst the highest in the world. To address this problem, we developed a participatory learning and action (PLA) intervention. Here we describe challenges and lessons learned while developing and testing this intervention in preparation for a large-scale One Health trial in northern Vietnam. We tested the PLA approach using community-led photography, and then reflected on how this approach worked in practice. We reviewed and discussed implementation documentation and developed and refined themes. Five main themes were identified related to challenges and lessons learned: understanding the local context, stakeholder relationship development, participant recruitment, building trust and motivation, and engagement with the topic of antibiotics and antimicrobial resistance (AMR). Partnerships with national and local authorities provided an important foundation for building relationships with communities, and enhanced visibility and credibility of activities. Partnership development required managing relationships, clarifying roles, and accommodating different management styles. When recruiting participants, we had to balance preferences for top-down and bottom-up approaches. Building trust and motivation took time and was challenged by limited study team presence in the community. Open discussions around expectations and appropriate incentives were re-visited throughout the process. Financial incentives provided initial motivation to participate, while less tangible benefits like collective knowledge, social connections, desire to help the community, and new skills, sustained longer-term motivation. Lack of awareness and perceived importance of the problem of AMR, affected initial motivation. Developing mutual understanding through use of common and simplified language helped when discussing the complexities of this topic. A sense of ownership emerged as the study progressed and participants understood more about AMR, how it related to their own concerns, and incorporated their own ideas into activities. PLA can be a powerful way of stimulating community action and bringing people together to tackle a common problem. Understanding the nuances of local power structures, and allowing time for stakeholder relationship development and consensus-building are important considerations when designing engagement projects.

## Introduction

Antibiotic resistance is a global public health problem that threatens modern medicine, and is projected to result in 10 million deaths a year by 2050 as well as $100 trillion USD cumulative economic costs if not tackled now (1). In 2019, an estimated 1.27 million deaths were attributed to bacterial antibiotic resistance (2). Resistance occurs naturally, but is amplified by the use of antibiotics for human and animal health, as well as environmental contamination through wastewater, sewage and manure (3). Between 2000 and 2010, worldwide antibiotic consumption increased by 35%, including large rises in use of last-resort antibiotic drugs, particularly in middle income countries (4).

Antibiotic resistance in Vietnam is amongst the highest in the world, driven by high levels of antibiotic use for both humans and animals (5). The use of antibiotics in farming accounts for 72% of total antibiotic consumption in Vietnam (6), and of the 28% used for humans, most antibiotics are used in the community, outside of hospital settings (7). Antibiotic sales from private pharmacies make up a large part of community antibiotic consumption (8) and 90% are without prescription (9).

Despite the large consumption of antibiotics outside of formal healthcare settings, the National Action Plan on Combatting Drug Resistance (NAP) 2013–2020 compiled by the Vietnam government (10), mainly focused on antibiotic stewardship and surveillance of antibiotic resistance in tertiary hospital settings. Although raising awareness around antimicrobial resistance (AMR) in the community was included in the NAP, there were no clear policies or targets related to this component, no attention to participation or collaboration, and it did not result in any significant community-level awareness or actions (11).

AMR has been described as a “super-wicked problem” because of the inherent complexity. There are numerous interrelated biological and social drivers, multiple local, national and international stakeholders across different policy sectors, and several conflicting goals that might each be reasonably pursued (12). The drivers of inappropriate antibiotic use are multifactorial, and a problem with complex and interrelated drivers requires complex social interventions, including components that seek to tackle antibiotic use in the community and farming.

Community participation is a major component of people-centered health systems (13), and is enshrined as a key principle in the Alma-Ata Declaration (14). Interventions that mobilize communities through participatory action-oriented approaches have been widely and successfully used to address complex social drivers of poor health outcomes for maternal and child health (15) and other health domains (13). These approaches work through active learning and collective problem-solving to change behaviors and social norms, and have been recommended by The World Health Organization (WHO) for promotion of maternal and newborn health (16). We use “participatory action-oriented approaches” as an umbrella term to discuss several related approaches, including participatory learning and action (PLA) (15), participatory action research (PAR) (17, 18), and community-based participatory research (CBPR) (19). These approaches are inspired by the work of Paulo Freire, Robert Chambers, and others (17, 20), and all have in common the aim of engaging community stakeholders in order to produce meaningful social change, using methods that can empower participants through a bottom-up approach to generate locally appropriate solutions (21). Generating local data for what is or is not working provides a powerful feedback loop, engenders a strong sense of ownership, and fosters an appreciation for the importance of evidence to inform decision-making. Participatory action-oriented approaches may work synergistically with education-based interventions to create sustainable, population-wide changes in knowledge and behavior.

Due to the low priority given to the community components of the NAP in Vietnam, few activities have been undertaken at this level, and there is a huge need to improve understanding and change behavior toward more appropriate use of antibiotics. Interventions that target antibiotic use in the community in other countries have mostly used passive health education approaches, through mass media campaigns, posters, leaflets and websites (22, 23). One Health interventions, working across disciplines to attain optimal health for people, animals, and the environment (24), have also mainly targeted leverage points that are low in the causal chain, without addressing the more distal drivers of the emergence and transmission of AMR, or the context within which antibiotics are used (25). Where there is a complex interplay of social, cultural and economic factors, active engagement of communities, farmers, and health professionals in solving problems may provide a more powerful way to stimulate action, accelerate behavior change, and create context-specific solutions, than simply increasing knowledge (14). Community engagement has been recognized as a promising method to win the fight against AMR because it empowers communities to look for solutions that best suit their context. WHO has recommended the use of community-based actions for raising awareness of AMR and changing behaviors (26), Wellcome Trust has developed a Responsive Dialogues approach (27, 28), participatory film projects have been used in Bangladesh and Nepal (29, 30), and other creative approaches to disseminating AMR knowledge to the public such as visual arts, museum collections, and science books have been implemented in the UK (31). A framework for community engagement for AMR identified sustaining and scaling up engagement interventions as a key challenge (32). A pilot study in Bangladesh using a community engagement approach to co-produce an intervention to tackle AMR reported that co-production of the intervention processes and materials with key stakeholders at policy, health system, and community levels and consideration of the health structure and socioeconomic and cultural context when designing the approach are needed to make the approach scalable (33). However, so far, there is limited evidence for the effectiveness of community engagement approaches toward appropriate antibiotic use compared to more traditional education-based approaches, or for understanding the pathways and facilitating conditions for successful behavior change using this approach.

To address the gaps we identified in the AMR agenda, including little attention paid to antibiotic use in the community, and the lack of evidence for the effectiveness of community engagement approaches to tackle AMR, we designed a multi-component One Health trial with collaboration across sectors. One of the intervention components involves PLA, and here we explore challenges and lessons learned from our formative research developing and testing this intervention component.

## Materials and Methods

### Study Population and Context

Vietnam's administrative structure is made up of 63 provinces, each divided into districts. Districts are further sub-divided into communes, which are made up of several villages. The People's Committee oversees governance at provincial-, district- and commune-level, and each commune has a People's Committee leader and a commune health center.

The research reported here was done in Vu Ban and Giao Thuy Districts of Nam Dinh Province. Nam Dinh is located in the Red River Delta, northern Vietnam, with high levels of antibiotic use in communities (34), and is a known hotspot for AMR (35). Nearly 80% of the population live in rural areas, with an average gross regional domestic product (GRDP) per capita of approximately $1,899 in 2020 (36). The Antimicrobial stewardship agenda had not reached the provincial or district hospitals in Nam Dinh, or into the lower-level commune health centers (37, 38). There had previously been research evaluating interventions targeting antibiotic prescribing in primary care in other districts in Nam Dinh Province (34), but no interventions targeting antibiotic consumers, and no interventions or awareness-raising activities at all in the two districts in which we worked. Target participants included primary caregivers of children under 5-years, women, and farmers. These populations were chosen to represent key groups with knowledge on antibiotic use and healthcare within households and farms.

The research partnership was between Oxford University Clinical Research Unit (OUCRU) (a registered non-profit, research institution in Vietnam), and the National institute of Hygiene and Epidemiology (a national-level health institution). In line with the government administrative structure, the study was managed by health institutions at the provincial-, district- and commune-level. For coordination and logistics, the OUCRU study team worked with all levels of administration. For implementing activities in the communities, the OUCRU study team worked directly with study participants at the village level. The study team included two supervisors, four research assistants, and a study coordinator. Two research assistants stayed in Nam Dinh, while the other two and the study coordinator stayed in Hanoi, and only traveled to the study areas for implementation activities. Prior to implementation, all staff attended a 1-week training on Participatory Action Research organized by OUCRU and PRAXIS UK, but for many of the team, this study was our first experience using the approach in practice. The study team had limited previous experience in planning and implementing such a large and complex study involving stakeholders from different governmental levels. All of the activities happened during the COVID-19 pandemic.

### Design

The study we report here was conducted during the development phase of a large-scale One Health trial, aiming to reduce antibiotic use and promote preventive behaviors in healthcare, community, and farm settings. The aim of this development phase study was to pilot a PLA approach and integrate community and partner ideas about using this approach into the development of an intervention for one component of the One Health trial. The lessons learned from this formative experience are reported in this paper.

Our participatory learning and action approach incorporated Photovoice methods as a tool to visually explore the issue of antibiotics and AMR in the community. The Photovoice study used a range of participatory tools and group activities, photo-taking, and discussions about the photos to facilitate exploration of current practices and understanding around antibiotic use and AMR. Photovoice was used as a method to stimulate discussions, provide insights into important issues, and as a medium for participants to share what they learnt with the wider community and raise awareness about AMR. Photovoice methods align well with the PLA approach, as Photovoice seeks to empower participants through active learning, using a bottom-up approach to generate community action (39, 40). However, as this was a formative research study, we did not have time to continue with PLA as an iterative process to support other community actions to tackle AMR.

We planned the study together with the National Institute of Hygiene and Epidemiology. They helped us to organize introduction meetings with local stakeholders from provincial, district, and commune levels, where we introduced the study's purposes and objectives and discussed the logistics. We started in November 2020 with the selection, by local government partners, of one commune from each of two districts in Nam Dinh Province. Two villages were selected from each commune by the study team (four villages in total). Women's groups were formed in two villages that had access to the commune health center and many pharmacies, and farmer's groups were formed in two villages that had livestock production and veterinary supply shops. A combination of purposive and self-selection sampling was used to identify a total of 26 participants across the four villages. The number of participants was determined by the number of cameras available. Participants were informed about the purpose and requirements of the study and gave their written consent to take part.

The Photovoice process consisted of seven meetings and a photo-taking period (Table 1). After the mass village meetings, a meeting was held to introduce participants to each other, share expectations and establish group ground rules. In this meeting, we introduced the topic of antibiotic use and AMR, discussed the situation in the community, and provided basic concepts of photovoice and photography techniques. Participants were asked to take photos capturing real situations related to antibiotic use in their community and in livestock production over a period of 2 weeks. After taking photos, the groups came together again and shared their experiences of taking photos and the issues they encountered related to antibiotic use and AMR. There were two rounds of photo selection: the first round was to individually choose favorite and topic-relevant photos and provide narratives; and the second one was to agree on core issues and themes and select photos for the exhibition. In the following meetings participants discussed how to share the stories about antibiotics and AMR with the wider community so that they could encourage appropriate antibiotic use and improve health in their communities. They developed a plan, organized, and held a community exhibition for sharing the photos and raising awareness about AMR. One exhibition combining the photos from women's and farmers' groups in one commune was held, and two separate exhibitions were held for women's and farmers' photos in the other commune. These were followed by wrap-up meetings to acknowledge participants' contributions, gather feedback on their experiences of taking part, and discuss potential follow-up and cooperation between participants, local stakeholders, and authorities to tackle the issue of AMR.

**Table 1:** Outline of meetings held in the Photovoice study.

**Meeting**	**Topic**	**Activities**
1	Mass village meeting	- Public gathering - Introduce the study to community members - Recruit participants
2	Introduction and camera training	- Get to know each other - Develop group ground rules and explore participants' expectations - Discuss the aim of the study - Screen video clips about the use of antibiotics and AMR in Vietnam and discuss the issues in their communities - The basics of the photovoice method - The basics of photography and camera usage - Discuss ethical considerations and consent when taking photos
3	Photo-taking (2-weeks)	- Take photographs of antibiotic use in the local community
4	Individual photo selection	- Share photo-taking experiences and issues encountered related to antibiotic use and AMR with the group - Individual review process: - Each participant reviews and writes narratives about their photos, and selects meaningful photos
5	Group photo selection	- Share selected photos and stories about antibiotic use and AMR with the group - Group agrees collective issues and themes - Group selects final photos and stories for exhibition
6	Exhibition plan	- Discuss how to share the stories about antibiotic use and AMR with the wider community to change behaviour and improve health - Discuss venue, time, visitors and invitations, refreshments, reception, other logistics issues - Plan the exhibition layout - Allocate tasks to team members
7	Exhibition	- Hold an exhibition to raise awareness about AMR in the local area
8	Wrap-up	- Present the study summary report to participants, stakeholders, and the local authority - Recognition of participation - Discuss how the results can be used by participants, and local authority to increase awareness about AMR and build a heathier community

### Data Sources and Development of Themes

We wanted to learn from the Photovoice study about how best to implement a PLA intervention to tackle AMR, so we held extended reflections and discussions about the process of implementing the study and summarized challenges and lessons learned. We made use of sources of information that arose during the process of development and implementation of activities, rather than transcripts from discussions and meetings themselves. These included field notes, photos, informal discussions, and observations, as well as participant, stakeholder, and partner feedback. After each activity, a report was written by the study team to summarize the activities and issues that arose. Reports were based on field notes, observations, and audio recordings of discussions. We also held regular debrief sessions after field activities, in which the study team reflected on implementation challenges and community engagement. Meetings were documented in minutes and meeting notes. Six researchers listed the main challenges and lessons learned that were identified in the above data sources regarding the development and implementation of the community engagement studies. One of the researchers categorized the challenges and lessons learned into preliminary themes. Then five researchers reviewed, discussed and revised the themes and definitions of each theme through a process of reflection and exchange over the course of 22 virtual meetings. Participants and partners were invited to provide feedback on the final themes identified as challenges and lessons learned. The final themes are presented below.

## Results

The challenges we identified were grouped into five main themes. These themes are summarized in Table 2, and include understanding the local context, stakeholder relationship development, participant recruitment, building trust and motivation, and engagement with the topic of antibiotics and AMR. For each theme we illustrate with examples and discuss the lessons learned that could be applied to address these challenges and enhance opportunities for listening and responding when scaling up this approach.

**Table 2:** Summary of challenges and lessons learned.

**Themes and sub-themes**	**Challenge**	**Lessons learned**
**Understanding the local context**		
Accessing background information	- Limited availability of health-related data about antibiotic use and AMR affected understanding of local context	- Gathering local information improves contextual understanding. Local partners can help to provide information that is not online, and where feasible, surveys and interviews can fill information gaps
Understanding local language and practice	- Study team not familiar with local terminologies and health-related or farming behaviours	- Learning context-specific terminologies, and developing a local vocabulary improves understanding and engagement
Understanding the context of partnership	- Study team not familiar with the local nuances of building relationships with partners	- It is important to understand local working dynamics and practices to build effective partnerships
**Stakeholder relationship development**		
Working with multiple stakeholders	- Navigating the power and culture dynamics of working with multiple stakeholders with different experiences and expectations can be complicated	- Multistakeholder partnerships have many advantages, including providing legitimate entry to communities and providing guidance and support for implementation of study activities - Feedback from different stakeholders can shed light on what did and did not work in the study, leading to stronger relationships and better implementation of future work
Balancing top-down and bottom-up approaches	- Top-down approaches are the norm for government partners, but are contrary to PLA approaches which require engagement and shared decision-making from the bottom-up	- It is important to take time to build consensus on the value of community engagement and using a bottom-up approach - In order to establish a trusting partnership with high-level stakeholders it may be necessary to strike a balance
Clarifying roles	- The involvement of different stakeholders in the local government management hierarchy proved to be more effective at specific stages of the study than at others - Most attention was given to encourage participation among community participants, and local stakeholders did not clearly understand the vision and methods of participatory research	- Taking time to listen and clarify roles - Identify at which stages of the study cycle each stakeholder should be involved - Involve local stakeholders in decision-making and establishing the shared vision so that they can be more actively involved
Recruiting local staff	- Lack of study team presence in the province and community make it difficult to develop partnerships - Difficult to find staff with both local knowledge and connections and experience of community engagement approaches	- Having local study team members helps to develop relationships with local partners and embeds the study in the community - Continuity of study team members has an impact on relationships with local partners and should be sustained where possible
**Participant recruitment**		
Recruiting participants	- Purposive sampling is the preferred approach for local and national partners, but is contrary to methods used for participatory learning and action (PLA) in which participants self-select to take part - Participation by self-selection was not always possible due to logistical and time-constraints	- For short-term projects purposive sampling is sufficient, but need to be aware of introducing possible biases - For longer-term engagement projects, taking the time and effort to negotiate and implement participation by self-selection is important - Working closely with local partners to build trust and explain the study and recruitment objectives is crucial for either approach
**Building trust and motivation**		
Establishing trust with participants	- It was difficult to establish trust due to limited personal interactions, lack of pre-existing connections with the community, and lack of understanding of local context - The study team lacked professional or technical expertise related to health or farming, and our organization was unknown in the study area	- Working with local stakeholders can provide access to their networks and facilitate personal connections - Working with local stakeholders who are trusted and have professional expertise can give credibility to establish relationships and legitimate entry into communities - Previous experience with projects and consent processes also have an important influence on trust
Building rapport between participants	- We needed to establish a safe and trusting environment among the members within each group to allow for open discussion	- It is useful to establish collective ground rules about how the group will work together and re-visit them throughout the process
Aligning expectations	- Misalignment of expectations in terms of what the study could deliver may have negatively affected motivation	- It is important to discuss participants' expectations and clarify any areas of misconception throughout the study
Maintaining motivation	- There were different levels of motivation and engagement between the groups related to local context, past experiences, competing priorities, recruitment processes, and disruptions - There was an expectation of financial incentives for participation, but this is not something that can be sustained for long-term participation, and creates a power imbalance - Participants expected non-financial incentives in the form of knowledge about health and medical care, but this was not included in our study activities	- It is important to explore and acknowledge differences in motivations and be flexible and responsive to these differences - Financial incentives may be useful for short-term engagement or specific activities - Discussion with participants and stakeholders about motivations for participation can provide ideas about suitable non-financial incentives - Incorporating training or health promotion sessions in engagement activities could provide a strong non-financial incentive for participation
Sustaining longer-term engagement	- Intangible benefits in the form of collective knowledge, social connections, skills, and confidence are less easy to communicate as benefits at the beginning of a study	- Intangible benefits may contribute the most toward sustained engagement in the longer term
Motivation during COVID-19 disruptions	- Movement restrictions dues to COVID-19 prevention measures disrupted some planned activities and made sustaining motivation challenging	- Listening to concerns and following participants' lead on when face-to-face activities could be held ensures participants are comfortable with planned activities - It is important to maintain relationships by keeping in touch about the situation and plans by phone and instant messaging
**Engagement with the topic of antibiotics and AMR**		
Understanding of AMR	- Limited knowledge about antibiotics and antibiotic resistance was a barrier to engaging with communities on this topic - Participants initially thought that overuse of antibiotics was not a threat, or that it was only a threat for other communities, and not relevant to them	- Careful consideration of local terminology and understanding makes communication clearer - Identifying pre-existing health concerns and showing how antibiotic overuse and resistance are related makes the issue more relevant - Sharing experiences related to antibiotic use revealed that there were impacts closer to home
Ownership	- The project was introduced to the community as a topic they didn't know very much about, but one that we wanted them to take the lead on	- A sense of ownership emerged as the study progressed and participants understood more about the issue and saw their ideas being incorporated into activities

### Understanding the Local Context

Prior to developing and implementing community studies, it is important to understand the local contexts in which the studies will take place. We encountered challenges related to accessing background information, local terminologies, and navigating the process of forming local partnerships.

We began the development of our study by conducting desk research to gather information about the situation related to antibiotic use and AMR in the study area, as well as to provide a general understanding of the economic, socio-cultural, and health contexts of the target populations. However, while some economic reports were available, it was hard to obtain government-published data and reports about the use of antibiotics and the context of AMR in the province. This led to a lack of understanding about the local context by the study team at the beginning of the study resulting in difficulty asking questions in the right way, or about the right things. For example, we were not aware that many pig farmers had lost their animals to recent bouts of swine fever and were no longer engaged in large-scale pig-farming. But this naivety also provided an opportunity for us to ask very basic questions about local healthcare and farming practices with genuine interest, and with fewer pre-conceptions about what we expected to find. We sought additional local information from our partners, and made use of our own research data on antibiotic knowledge and use to fill the information gaps.

While all researchers who worked with the communities spoke Vietnamese, there were some local terms and nuances they did not understand. Misunderstandings may have influenced initial levels of engagement and our ability to communicate meaningfully with participants. To solve the language problem, we developed a local vocabulary through our discussions with participants, including terms relevant to daily life as well as AMR. This local vocabulary also gave us insights into people's behavior. For example, we understood there was an expectation to receive drugs for treatment when people go to primary healthcare centers, because they usually said, “they go to ask for medicine” instead of, “they go to see the doctor.” Through our discussions and engagement with participants, we gathered more local information and learned context-specific terminologies related to antimicrobial treatment and healthcare-seeking behaviors. It took time to develop this contextual understanding, but it helped us to communicate better and develop more suitable engagement approaches and messages.

At the partnership level, the dynamics of working relationships are highly influenced by historical, political, and cultural contexts, but these are rarely documented or explicit. The study team initially lacked understanding of the communication and operational structures of the health system. An example of this was our underestimation of the importance of dining with partners as way to establish relationships. Being bounded by our organizational and funding structure meant that we missed this opportunity to build rapport with our partners at the initiation of the study. Not understanding the local nuances of working relationships made it difficult to establish partnerships and efficient cooperation.

### Stakeholder Relationship Development

Antimicrobial resistance is a complex issue demanding cooperation between multiple sectors as well as vertical coordination between national and grassroots levels. There is a longstanding relationship between OUCRU and the National Institute of Hygiene and Epidemiology, but in order to engage with communities we had to establish new relationships with local authorities inside and outside the health system, at provincial, district, and commune levels in the study area, where neither partner had existing relationships. We encountered challenges related to how to build strong relationships including working effectively with multiple stakeholders, balancing top-down and bottom-up working styles, clarifying roles, and recruitment and retention of local staff with the right combination of skills to coordinate study activities.

Our aim was to engage with community members, and to reach communities, we had to work with multiple stakeholders. Navigating the power and culture dynamics of working with multiple stakeholders required paying attention to each partner's experiences and expectations. Stakeholder preferences about study design and communication, as well as their experience working with the community varied. For example, we planned informal and interactive introduction meetings to create a friendly atmosphere, but this gave the sense that we were unprofessional, and we were advised afterwards to follow a more formal format in such meetings. In general, national-, provincial-, and district-level stakeholders preferred more hierarchical top-down management and formal communication. Meanwhile community-level stakeholders valued familiarity and kin connections and were more comfortable with informal interactions. Local stakeholders provided legitimate entry to communities and provided guidance and support on implementation of study activities. Feedback from different stakeholders provided different perspectives and ideas for improving implementation of future work.

The top-down approach preferred by government partners enabled decisions about study implementation to be transferred smoothly from national-level partners to local stakeholders, and gave us the credibility to establish a relationship with local authorities. On the other hand, the preference for top-down management created a challenge in using a PLA approach, which requires bottom-up engagement, involving stakeholders in the study development and implementation process. The prevailing top-down working style made it difficult to encourage an active role in research to those who were more familiar with being involved passively, and they found the bottom-up-approach complicated and time-consuming. It took time to build consensus about using a bottom-up approach, and in order to establish a trusting partnership with national and local partners we had to strike a balance between top-down and bottom-up approaches.

We discussed the breadth and depth of participation with partners through open dialogues to share ideas about the research objectives, ethics, engagement method, logistics, and expected outcomes. This continuous cycle of communication occurred over a period of time, with some disruptions and delays due to administrative processes and COVID-19. The involvement of different stakeholders in the management hierarchy proved to be more effective at some stages of the study than at others. For example, it was necessary to have interactive participation of local stakeholders at every stage in the community engagement process, while stakeholders at national and/or provincial level could participate in the consultative process during the introduction and evaluation phases more than the implementation period. We encouraged shared decision-making with partners throughout the research process, but it was not always successful. We informed partners of our plans and listened to their suggestions and feedback, but they did not clearly understand the participatory method and our vision for using this method for the study, so their participation was passive and mostly took the form of responding to our questions and requests. On reflection, we had failed to engage with local authorities as equal research partners and found that determination of how much involvement and participation was required on what issues and at what stages should be carefully thought through.

Our lack of presence in the study area made the formation of personal relationships with local partners and study communities challenging. This arose due to difficulties recruiting and retaining qualified local study staff so that most activities were managed from Hanoi, and was further exacerbated when staff outside the study area were not able to travel due to COVID-19 restrictions.We tried to recruit local staff who understood the local context, culture, and language, and were already well-connected with local authorities and communities. In order to liaise between the research organization and local stakeholders, a combination of skills in community engagement, facilitation, diplomacy, and project management are required, as well as the ability to communicate in both Vietnamese (local language) and English (language of the research organization). However, it was difficult to identify local candidates with the right combination of skills, and desired candidates were more likely to be based in larger cities and were reluctant to relocate to the provincial town. A high staff turnover in this position caused negative effects on partnership development, due to different working styles, disrupted communication, and difficulty establishing a stable working relationship with partners.

### Participant Recruitment

Challenges in recruiting participants were related to achieving the right balance between different approaches, each approach having advantages and disadvantages (Table 3). Local partners preferred to assign participants, but participants in PLA activities usually self-select or volunteer to take part, and this was considered a pre-requisite to develop a sense of ownership of the study activities. In the Photovoice study, participation over several months was required, and we wanted participants to have the opportunity to volunteer (self-selection sampling), rather than be assigned by local authorities. Consultations were held with local authority representatives and mass meetings were held in three villages to introduce the study, explain what would be involved for participants, and invite volunteers to take part. Seven participants volunteered from each village, making 21 in total. In the fourth village, it was not possible to hold a large gathering, therefore local commune officers purposively selected five participants. These participants were also informed about the study requirements and gave their written consent to participate. Here we outline the challenges and opportunities of both purposive and self-selection sampling methods.

**Table 3:** Summary of advantages and disadvantages of different sampling approaches.

	**Purposive sampling**	**Self-selection sampling**
Management style	- In line with top-down management, which is more in tune with local government approaches	- In line with bottom-up management, which is more in-tune with PLA approaches
Partnership	- Provided an opportunity to develop relationships with local stakeholders, and provided official endorsement for the study	- Enabled better development of relationships with communities and participants
Logistics	- Quicker and simpler for local partners to implement	- Required more time and resources to organise mass recruitment meetings
Recruitment	- The right number of participants were recruited	- Had to plan for the possibility that too many or too few participants would volunteer
Selection	- Introduced bias, such that participants did not reflect the population we wanted to engage	- Participants were hesitant to join due to lack of familiarity with the organisation, lack of perceived relevance of AMR, and perceived lack of personal capacity
Engagement	- Participants were less flexible about meeting times - Some participants did not fully participate or contributed little to discussions	- Flexibility on time and duration of meetings - Dynamic discussions with involvement of all participants
Outcomes	- Photos and narratives were more superficial and did not explore the topic in depth	- Photos and narratives captured thoughtful stories about the topic

#### Purposive Sampling

Purposive sampling has several advantages, including being quick and simple, and allowing local partners to recruit the most “qualified” participants. They proposed that these participants would benefit the study the most and also act as ambassadors providing positive reflections of the community. For the purposively sampled farmers' group, the local commune authorities were provided with information on the nature of the study and recruitment criteria. The local authorities proposed adding selection criteria, including good communication skills and experience, so that the participants would perform better and produce better study outcomes. Although the study team preferred to keep minimal exclusion criteria, the local authorities may have consciously or unconsciously applied their own. With such open criteria, among numerous eligible candidates, individuals with a personal relationship, position associated with their profession, or involvement in government bodies might have a higher chance of being selected. For example, among five of the assigned farmer participants, three farmers were commune officials and two others were heads of farmer groups in two villages. Although all of them met the recruitment criteria, they did not represent the general population well and their levels of motivation and engagement differed from self-selected participants (see Building trust and motivation below).

Using the locally accepted recruitment approach provided an opportunity to develop relationships with local partners, by “doing things their way.” Through this, we acknowledged the valuable knowledge of local partners about the community. As a new organization in the area, having the local authorities recruit participants helped to alleviate people's suspicions, and enhance the perception and credibility of the study. To reduce bias with purposive sampling, and obtain more generalizable results, it is crucial to work closely with local partners to develop mutual trust, explain study objectives, and clarify recruitment criteria.

#### Self-Selection Sampling

Self-selection sampling can take longer than purposive sampling and have a higher risk of failure to identify participants. There were practical challenges to the success of this non-coercive recruitment process, and here we outline two of these.

Firstly, this approach required the study team to invest more time, resources, and preparation as we needed to organize mass meetings to introduce the research and recruit participants instead of relying on the local partners. Moreover, we had to plan for two possible scenarios: not recruiting enough participants through the mass meetings to ensure good group dynamics; or having too many people who wanted to join and managing disappointment.

Secondly, people were hesitant to volunteer for this study, and we identified three main reasons for this: guardedness about working with a new organization, perceived lack of relevance of AMR, and perceptions of their own personal capacity. People in one village were initially suspicious of us as strangers in the area (see Establishing trust with participants below). People who thought the research did not resonate with or provide direct health benefits to them, their family, or their community, doubted its relevance, and were reluctant to take part (see Engagement with the topic of antibiotics and AMR below). Furthermore, potential participants were hesitant to believe in their own capacity to provide valuable input or expertise. Those with little or no educational background were reluctant to volunteer, although the inclusion criteria clearly highlighted that there was no requirement related to personal qualifications.

Overall, purposive sampling was sufficient for short-term participation, but self-selection was more appropriate when intense participation and commitment over a longer period was required, and there were advantages and disadvantages of both approaches (Table 3). The level of engagement and participation was higher from the self-selected groups than purposively selected. For example, the dynamics in the discussion were much easier in the self-selected groups and they did not worry about the time/length of the meeting. There was less flexibility in meeting with the farmer's group that was assigned, as their main source of income was salaried employment not farming, and they had other work to do. Several of these farmers did not fully participate in the discussions, contributed little, and were less enthusiastic about the study topic. Both self-selected and purposively selected participants took similar numbers of photos, but the topics were different. Participants in self-selected groups took photos and wrote narratives that contained more thoughtful stories about inappropriate antibiotic use. These self-selected farmers were owners of big farms, so they had decades of experience in livestock production and were more reflective of the issues.

### Building Trust and Motivation With the Participants

We identified six sub-themes related to trust and motivation, including establishing trust with participants, building rapport between participants, aligning expectations, maintaining motivation, sustaining longer-term engagement, and motivation during COVID-19 disruptions. Issues around trust and motivation also related to our initial lack of understanding about the local context (as previously discussed) and engagement with the topic of antibiotics and AMR (discussed in the following section).

#### Establishing Trust With Participants

We were able to leverage the pre-existing relationships and trust that participants already had with local authorities and partners. Mass meetings were endorsed and attended by local authorities, or purposively selected participants were invited by local authorities. As part of the consent process, participants were provided with information about the study, and informed about the ethical approval by a national-level institution, which further extended their trust. To reinforce their initial acceptance and build personal trust, we took time to build rapport and create an open and comfortable atmosphere for sharing ideas at the beginning of each discussion with general conversation and interactive activities. This approach allowed rapport to evolve at a pace the participants were comfortable with.

The main challenges related to establishing trust between the research team and participants were that there were limited pre-existing connections with the community, and there were limited personal interactions due to a lack of study team presence in the area and movement restrictions due to COVID-19. We were not able to spend enough time with and in the study communities to become involved and establish strong trust with them, and we had to rely on local partners to maintain interactions. Our organization is recognized globally for health-related expertise, but participants had not heard of the organization. Additionally, the team did not possess specific professional or technical qualifications related to health or farming that gave us independent credibility, so we relied on our local partners to lend credibility to our study and provide access to their networks. We then gradually built up trusting personal relationships.

Previous experience with fraudulent projects negatively affected trust, in one village. These previous experiences made local authorities and communities suspicious of outsiders, and we had to work harder to recruit participants from the public meeting, and also to build trust with the women's group in that village. It seemed to be easier to build trust with farmers, as animal health was a less sensitive issue than human health, and perhaps less prone to confidence trickery. However, one farmers' group was also assigned to participate, and for this group their trust was an extension of their trust in the local authorities who assigned them. Consent and permission also played a role in establishing trust and were sought before recording any discussions, making materials public. Trust was further developed as we delivered on the study objectives we had outlined and shared photos and stories in a meaningful way with the wider community.

#### Building Rapport Between Participants

PLA usually requires participation during a series of meetings over several months. In the Photovoice study we needed first to establish a safe and trusting environment, and then build social connections among the participants within each group to allow for open discussion during meetings. Although participants lived in the same village and already knew each other, their relationships were not close enough to make them feel comfortable to share their perspectives about antibiotic use behaviors, especially inappropriate practices. This applied to both women's and farmers' groups.

The series of meetings began with group formation, exploration of participants' expectations, and establishing ground rules. Each group established a set of ground rules which was agreed by all group members. Ground rules differed between groups, but key principles included confidentiality, being respectful, listening to each other, and having an encouraging and learning attitude. Throughout the implementation period, group discussions, and teamwork activities were conducted in adherence to group ground rules, which helped to reinforce trust between group members. Participants' perspectives were considered equal, with no voice carrying more weight than others during the decision-making process.

#### Aligning Expectations

It was important to discuss participants' expectations and clarify misconceptions at all stages of the study, in order to maintain trust and motivation. To better understand individuals' needs and to facilitate participants working toward shared goals, we explored personal and group expectations. Expectations did not always align with what we were able to deliver, which may have affected motivation (Table 4). For example, some participants expected a professional training on health promotion or good farming practices. This was particularly the case for women's groups, who wanted specific guidance on which antibiotics to use for which illnesses when their families were sick. At the start, we explained that we could not offer this kind of training. Some participants remained motivated to work with us, but not all participants remembered this point, and we had to re-visit their expectations during implementation. We explained how participation could help them and their communities to learn more about antibiotics and antibiotic resistance through a process of shared learning rather than a one-off training session. Farmers had a lot of farming experience and seemed to be more satisfied with exchanging ideas about farming practices with each other. During the wrap-up meeting we reviewed the expectation list and evaluated what they received and what we had achieved together. A continuous process of explaining and addressing misconceptions in expectations enhanced engagement and cooperation.

**Table 4:** Participant and study team expectations from the Photovoice study.

	**Participant expectations**	**Study team expectations**
Personal expectation	To be capable of taking (a lot of) nice photos	Participants to be able to use a camera and take photos related to experiences of antibiotic use
	To socialize with other community members	Participants would share opinions openly and work together well
	To learn from others	Participants to listen to and learn from each other about antibiotic use in the community
	To learn from experts and/or health professionals about antibiotic use, negative impacts, and risks of antibiotic resistance	Study team to provide a basic introduction to the topic of antibiotics and antibiotic resistance
	To know when and how to take medicine correctly	Not included
	To learn good animal husbandry practices	Not included
	To prevent diseases and have better care for domestic animals	Not included
	No particular expectation, just simply want to participate	To recruit participants who would be engaged and motivated
Inter-personal expectation	To share experiences and lessons about antibiotics and common illnesses with family and friends	Participants would share information with their family members, friends, and neighbors
	To help the community to become knowledgeable about antibiotics and prevent antibiotic resistance	To organize an exhibition to share the issues with the wider community
	To encourage other farmers to pay more attention to meat safety and clean livestock management practices	Not included

#### Maintaining Motivation

Maintaining motivation was an ongoing process which required adapting the study design to listen and respond to the community effectively. The most visible indication of lack of sustained engagement was the drop-out of participants in the middle of the study (5 of 26 dropped out). The main reasons for drop-out were not related to trust or motivation, but to competing priorities, such as personal and family issues. In most cases, these activities were not something the study could or necessarily should compete with. But, in order to sustain participation and minimize conflicting engagements, we made the meetings as convenient as possible for participants, by arranging them at times and locations decided by them, to fit into their schedules. This often meant that meetings were scheduled late in the evening or on the weekend. There were different levels of motivation and engagement between the groups, and this was influenced by different prior experience with external projects, different socioeconomic contexts, personal priorities, the sampling strategy, and disruptions to planned activities. Being flexible and responsive to these differences was important.

We think that when deciding whether to take part and to continue to participate, participants had to balance the costs and benefits. Their decisions may have been influenced by incentives and other perceived study benefits, prior expectations about payments from international organizations, as well as the participants' competing priorities, value of their time (e.g., in the form of lost income opportunities), and socio-economic background. We provided financial incentives in the form of reimbursements for transport and time during activities. Some participants said they were reluctant to attend activities unless there were financial incentives, or they only took part in meetings peripherally until they received reimbursements. Financial incentives also acted as a bond with the study. This bond may have encouraged participants to engage in the activities, but also signified a commitment. One farmer returned the reimbursement when he withdrew from the study, explaining that he had broken his commitment and did not deserve the incentive. The importance of financial incentives as a motivation for participation differed between communities, and we hypothesized that this was due to different selection processes, prior study experiences, as well as socioeconomic and other contextual differences.

There were disagreements about incentives between the study team and local and national partners. We listened and asked for advice from our partners and participants, to understand the different perspectives and how to improve motivation and engagement overall. For some participants, financial incentives were expected and were the strongest motivator for their participation. In addition to financial incentives, the community expected to get some non-financial benefits, for example information or knowledge about health or livestock management. They did not expect to benefit from the longer-term goals of the study, to improve community health and save lives from AMR, because this pilot study had limited scope for intervention. Information or training sessions could have been used to provide short-term non-financial incentives to participate, in addition to or instead of financial incentives.

#### Sustaining Longer-Term Engagement

Apart from the financial incentives, there were few tangible benefits participants gained during the project, and this may have influenced their engagement. However, there were several intangible benefits that included knowledge gained through learning from each other, confidence and skills in taking photos and communicating stories, social connections and solidarity, and helping their community. These intangible benefits emerged slowly, but over time became apparent to participants and were important for sustaining engagement in the longer term, even in the absence of other financial or non-financial incentives.

#### Motivation During COVID-19

Disruptions to activities caused by the COVID-19 pandemic created additional challenges to sustaining motivation. Many of our activities were delayed as the study team was not able to visit the communities regularly due to movement restrictions. This led to some loss of engagement, difficulty sustaining rapport and personal connections, and forgetting about the remaining study activities after long breaks. In the Photovoice study, most of the activities were held between November 2020 and April 2021, but the final community exhibitions were held for two groups over 12-months after the last meeting in which preparations were made. To maintain motivation throughout these disruptions, we listened to participants' concerns about the changing situation and local context of COVID-19, what means of communication they preferred, and when and how they were comfortable to hold face-to-face activities. We tried to maintain our relationships by keeping in contact with participants by phone or instant messaging app to keep up-to-date about the situation and plans. However, there was a noticeable loss of interest in the project after the long delay.

### Engagement With the Topic of Antibiotics and AMR

#### Understanding of AMR

Narratives from photographs and discussions showed that participants had ambiguous concepts about what antibiotics are and how they should be used, and had been given little information from health-workers. Farmers seemed to have more knowledge about antibiotics and AMR, as they often received training from companies, and were motivated, self-guided learners, because improving farming practices had direct benefits for their livelihoods and profits. Participants were familiar with the general concept of drug resistance rather than antibiotic resistance, and felt worried about it, but had limited understanding of what either term really means. However, neither women nor farmers thought that AMR was an issue affecting their community. Participants knew that self-medication without seeing a doctor is not recommended, but in practice many did this. Farmers knew about AMR and laws prohibiting the use of antibiotics in animal feeds, but they believed antibiotic products may still be present in some unlabeled products. Due to the lack of local veterinary services, farmers used their own experience and knowledge, and bought animal medicines and vaccines to administer themselves, sometimes including leftover human antibiotics. The study team was cautious with the use of technical and academic words. Community interest was negatively affected when our communication resources contained specialized language and terms that did not fit local understanding. To enable us to listen and respond effectively, we used the local vocabulary we developed (see Understanding the Local Context above). We also consulted local partners and community participants to help us to simplify the language and concepts related to AMR in our future intervention materials.

Participants were interested in health and interventions to improve their health, but their main health concerns were chronic diseases and perinatal and nutritional disorders in children. For those that understood AMR, this was not perceived to be important for them or their community, and they felt it did not affect their life in an obvious way. Participants initially thought that overuse of antibiotics was only a threat for other communities, but after sharing their experiences, they discovered that there were impacts closer to home. One mother shared the experience of side-effects her child had had after antibiotic injections, another shared how her child's teeth had been damaged from taking antibiotics, and a farmer shared how she used to use leftover human antibiotics for her chickens. We tried to make AMR more tangible for participants by connecting their health concerns with the inappropriate use of antibiotics and AMR. We talked about their specific health concerns, and then we slowly attached our study and AMR issues to these. This helped them to recognize that antibiotic overuse and resistance and other health issues are inter-connected.

#### Ownership

The study was introduced to the community as a health project about a topic that they did not know very much about, but one that we wanted them to take the lead in finding solutions for. In order to establish a sense of ownership, we informed participants clearly from the beginning about the importance of their participation and their ownership of the outcomes. We encouraged participants to play a dynamic role and take the role of co-researchers, rather than objects of study. Participants were informed that they would use their photos and stories to raise awareness about the problem of overuse of antibiotics and AMR in their own and other communities. Participants developed a sense of ownership as the study progressed and this further contributed to their sustained engagement. As participants understood the issues more and how they were relevant to their lives, and incorporated their own ideas into the implementation of activities, they began to somewhat see the products of the study as their own (rather than the study itself which was initiated by us) and they were more motivated to contribute their time. Exhibitions were an unfamiliar concept in these communities, and participants might have chosen to share their stories and raise awareness in the wider community about antibiotics and AMR in other ways if the project had allowed for this, but participants were still excited and proud to co-organize the photo. Despite their engagement and ownership of the activities, at the end of the project, participants still did not think that AMR was a tangible problem or a high priority in their community compared to other issues. In particular, many farmers said they didn't use antibiotics, they followed guidelines, used vaccines to prevent animal illnesses, and their farming was profitable, so they were not worried about AMR.

## Discussion

We identified five themes related to the challenges of implementing community engagement projects related to antibiotic resistance. These included: understanding the local context, stakeholder relationship development, participant recruitment, building trust and motivation, and engagement with the topic of antibiotics and AMR. Similar themes have been cited in literature on community engagement, such as multistakeholder partnerships (41–44), trust (45–48), and the nature of participation and hierarchies in participatory action-oriented approaches (49–51).

Other scholars concur that understanding context, in the form of local agendas, culture, expertise, and organizational structure and process, form a basis to establish trust, respect and fuel further collaboration with partners in participatory research (52, 53). We found that it was difficult to establish connections with local stakeholders at a personal level through non-research activities due to our lack of contextual understanding, and this affected their trust and support during study implementation. This resonates with research on business culture in Vietnam indicating that bonding with partners through non-business activities such as feasts and banquets can foster collaboration (54). A culture-centered approach, that honors community knowledge in research design and implementation, can ensure integration of community voice and agency in health education interventions and can result in more structural change (55).

Partnership development between academic and community partners is an important component of participatory action-oriented approaches, and requires investment in team building, sharing resources, and mutually exchanging ideas and expertise. Incorporating feedback from different stakeholders can lead to stronger relationships and better implementation of future work (56). Partnership with local stakeholders is key to establishing local ownership and longer-term commitment and sustainability (14). However, the Vietnamese public sector has a very strong top-down management style (57), and partnerships with local government meant that we had to incorporate some elements of top-down approaches which conflicted with the bottom-up approaches and shared decision-making required for PLA. The traditional top-down management style also created a power imbalance between our team as researchers and our local partners. Suggestions to address these power dynamics in community-engaged research include understanding of context, having a shared vision, and inspiring leadership, diversifying partners for their expertise (53), and establishing ground rules to ensure all partners, including researchers are clear on their roles and equal in decision-making in all study phases (42). We focused on encouraging participation and shared decision-making among participants, but needed to invest more effort to reach a shared vision with local authorities and involve them as equal research partners in all study stages. In agreement with other practitioners, we found that the development of a collaborative partnership is crucial, but it takes time to build consensus and mutual agreement on study goals as well as emphasize the value of community engagement and using a bottom-up approach (58, 59).

The philosophy and methods used in participatory community development emphasize the importance of using a bottom-up approach to participant recruitment (60, 61). Allowing participants to volunteer or self-select can promote recruitment of participants who have little visibility but share common interests in healthcare issues and are motivated toward making changes in their community (62). In our study, self-selection sampling through public gatherings proved to be a good opportunity to introduce the research team and research activities to a large population. However, we had to employ a purposive sampling approach through local authorities to recruit participants in some activities due to time limitations and partner preference, which conflicts with the bottom-up approach required for engagement and participation. Research from the south of Vietnam reported that using a top-down purposive sampling approach may arouse concerns that participants who are assigned may feel coerced to join, and agree to participate without being well-informed about the study activities and/or their roles and benefits, which may in-turn lead to lower motivation and higher likelihood of dropping out (63). However, misunderstanding study requirements, risks and benefits may equally apply to those who self-select to take part, and early clarification of misconceptions is important to ensure continued participation. Working closely with local partners and building trust is an important prerequisite regardless of the recruitment approach.

Although establishing trust and sustaining motivation has been recognized as essential for participatory action-oriented approaches to be successful (45, 46, 48, 64), it is hard to evaluate trust and trustworthiness (47). Echoing this, we found it difficult to know whether or not we had gained trust from our local partners amid the challenges we faced in communication and study implementation. We recognized our shortcomings in cultural understanding and study management skills as a challenge to establishing our trustworthiness. We were received with some level of trust when we were introduced to community members by people they trusted, such as local authorities or trusted members of the community. However, as trust is a multi-dimensional construct (47), we found that this “abstract trust,” though helpful for partnership establishment, was not meaningful enough for engagement. Concerns about safety and confidentiality prevented participants from opening-up and engaging. Therefore, it is important to create an “institutional trustworthiness” focusing on bidirectional communication for listening and addressing concerns (48). Another means for studies to establish trust is to demonstrate their good intentions by providing material benefits such as money, health resources, or farming inputs for community participants (65), but the PLA approach focuses on building capacity rather than providing inputs, so this was not considered to be appropriate in our study. Time and effort was required to create rapport and common understanding and establish trust. Our partnership with national and provincial institutions lent credibility to the study and provided access to local networks at the beginning, but establishing trust directly with participants and gaining their support for the PLA approach, required development of mutual understanding about the research methods and agreement on shared principles. Reaffirmation from the local authority, continuous communication, honest explanation, and recognition of the community's priorities were key elements that helped us build up mutual trust gradually.

Managing motivation and expectations, and when and how to use incentives, are recurring themes in community participation (45, 46, 66). The discussion of incentives raises complex ethical questions about how we can give something back to participants without incentives being seen as coercive (67). Financial incentives may increase participation in research (68), but are not a sustainable means to secure long-term participation and engagement, or to enhance a sense of local ownership of the change process. Giving incentives creates a transactional relationship between the research organization and the community, which has an inherent imbalance of power. But the use of incentives has become standard practice, and sets a precedent for future projects, particularly for international non-governmental organizations (66). This practice can unintentionally become an obstacle to shifting participants' motivation for participation from individual toward community benefits. When there are financial incentives tied to participation, this may also influence who local partners select to participate, perhaps prioritizing their relatives or friends, and creating a sense of nepotism. In our study, we had to decide what type and what level of incentive was possible, desirable, and appropriate. Incentives had both positive and negative effects on participants' motivation, and it was important to balance these to successfully implement community-led activities. On the one hand, incentives encouraged participants to take part in our activities by giving them some financial benefits, motivating them to spend their time on our study, and signifying their commitment to participate. Since participants did not receive any other material benefit for their participation, incentives in the form of cash payments or in-kind payments or gifts were easy tokens for their participation, and may have been enough for short-term engagement. On the other hand, PLA requires engagement over a period of time, and participants may expect and deserve more for their participation. When financial benefits were the main motivation, participants dropped out or did not fully engage. Listening to partners and participants helps develop understanding about norms and motivations. Other researchers concur that longer-term, non-financial incentives or benefits, may include collective knowledge, social connections, skills, and confidence (67). These benefits can be difficult to explain, but may become clearer as the study progresses, and serve to sustain engagement over time. The wider community benefits, such as improved community health, should also be explained, but may only become apparent much later. Financial incentives are a sensitive issue to discuss, but it is important to listen to participants, and understand the implications of different approaches.

It has been widely documented as a barrier to addressing AMR in communities that antibiotics are not clearly recognized and the concept of AMR is not well understood (69, 70). In our study we found that there was low awareness and low perceived importance of AMR in the community, and this made engagement on the issue particularly challenging. Agendas for community-based research or development work have often been criticized for following NGOs'/governments'/funders' interests instead of being based on mutual decisions made with communities (49, 51, 71, 72). We found this argument spoke to us as we struggled to integrate the topic of antimicrobial resistance, which appeals to researchers and funders as an urgent problem, but was not perceived by communities as a major concern. Limited population-targeted messages about antibiotic use and AMR meant that the problem was not visible or prioritized in the communities, there was a lack of interest in participating in study activities, and lack of motivation for change. This situation illustrated a conflict between research and donor interests that will be a challenge in ensuring the methodology of PLA remains true to its purposes when it is scaled up or applied elsewhere.

This study had some limitations. Using Photovoice methods to test the PLA approach gave us the opportunity to learn about stakeholder relationship development, group formation, trust and motivation, and engagement on the topic of AMR, which have informed the development of a larger scale PLA intervention. Participants had lots of ideas to improve and scale-up intervention activities in their communities, but this study mainly focused on the active learning phase of PLA, discussing problems and their causes. Photo exhibitions to raise awareness about AMR were the only community-led actions, and participants did not have a chance to develop their own strategies to tackle AMR. Thus, we cannot apply our findings to the whole PLA cycle. This study also represents experiences from one province and a few communities, and experiences may vary depending on the context and local personalities involved. There were some areas of engagement in which women and farmers may have differed, but with only four groups, it was difficult to tell whether this was because of differences related to human and animal antibiotic use, the fact that one of the farmers' groups was assigned, or other contextual differences between the communities. The themes presented here were refined by the study team, reviewed by our national partners, and a summary was discussed with local partners and study participants, however, the views presented here may be biased toward our own perspective. Due to disruptions caused by COVID-19, some activities were not completed in one community at the time of writing, so we were not able to draw our findings from the full scope of study implementation. These disruptions also affected some aspects of implementation and engagement.

In conclusion, the development of effective partnerships and community engagement is complex. Building relationships, developing contextual understanding, and implementing participatory approaches takes time, which is sometimes beyond the scope of short-term research funding, but is important for sustaining motivation and longer-term engagement. AMR was our research agenda, but was a topic that the participants in our study did not know very much about, and it was challenging to gain their interest. A sense of ownership emerged as the study progressed and participants understood more about the issue, shared experiences that illustrated how antibiotics and antibiotic resistance affected people they knew, and saw their ideas being incorporated into activities. These lessons will be important for our upcoming One Health trial, and other participatory action-oriented approaches to address AMR.

## Data Availability Statement

The raw data supporting the conclusions of this article will be made available by the authors, without undue reservation.

## Ethics Statement

The studies involving human participants were reviewed and approved by National Institute of Hygiene and Epidemiology Institutional Review Board in Biomedical Research (IRB-VN01057), and Oxford Tropical Research Ethics Committee, University of Oxford (Reference 529-19). The patients/participants provided their written informed consent to participate in this study.

## Author Contributions

SL and TP conceived of the study and were the Principal Investigators. HT designed the community-led media study component with AL. HT, HC, PB, YN, AL, GV, TT, and HN implemented the study activities. HC, HT, and SL conceived of the article. HC categorized challenges and lessons learned into preliminary themes. HN, YN, GV, TT, SL, and JV reviewed themes. JV oversaw review of themes. All authors contributed to the content, reviewed drafts, and approved the submitted version.

## Funding

This research was funded through a Medical Research Council UK public health intervention development award (MR/S001964/1) and a Wellcome Trust public engagement seed award for the Oxford University Clinical Research Unit as part of Wellcome Africa Asia Programme funding (106680/Z/14/Z). Open access publication fees are also provided Wellcome Africa Asia Programme funding.

## Conflict of Interest

The authors declare that the research was conducted in the absence of any commercial or financial relationships that could be construed as a potential conflict of interest.

## Publisher's Note

All claims expressed in this article are solely those of the authors and do not necessarily represent those of their affiliated organizations, or those of the publisher, the editors and the reviewers. Any product that may be evaluated in this article, or claim that may be made by its manufacturer, is not guaranteed or endorsed by the publisher.
